# Supplementary material for: Mapping factors that may influence attrition and retention of midwives: a scoping review protocol
Source: BMJ Open. 2023 Oct 20;13(10):e076686. doi: 10.1136/bmjopen-2023-076686 (PMC10603492; doi:10.1136/bmjopen-2023-076686)
Supplement: Supplementary data [file bmjopen-2023-076686supp001.pdf]

Database searches

Table 1. CINAHL search terms

Search ID	Search terms	Results
S1	(MH "Midwives+") OR (MH "Midwife Attitudes") OR (MH "Midwifery+") OR (MH "Nurse Midwifery") OR (MH "Midwifery Service+") OR (MH "Nurse-Midwifery Service") OR (MH "Maternal Health Services+")	53,880
S2	TI (midwi* or (maternity N3 service*) OR AB (midwi* or (maternity N3 service*)	41,052
S3	(MH "Personnel Retention") OR (MH "Personnel Loyalty") OR (MH "Employment Termination")	15,649
S4	TI (work* or profession* or employ* or occupation* or role* or organisation* or position or career* or vacanc*) N5 (retention or retain* or remain* or stay* or leav* or quit* or resign* or attrition or turnover) OR AB (work* or profession* or employ* or or occupation* or role* or organisation* or position or career* or vacanc*) N5 (retention or retain* or remain* or stay* or leav* or quit* or resign* or attrition or turnover)	23,722
S5	S3 OR S4	37,028
S6	S1 OR S2	57,065
S7	S5 AND S6	429

Table 2. MEDLINE search terms

Search ID	Search terms	Results
S1	Midwifery/	21300
S2	Nurse Midwives/	7545
S3	(exp Maternal Health Services/	57972
S4	Midwi*.tw.	29057
S5	(maternity adj3 service*).tw.	2735
S6	1 or 2 or 3 or 4 or 5	91332
S7	personnel loyalty/ or personnel turnover/ or work engagement/	8644
S8	((work* or profession* or employ* or occupation* or role* or organisation* or position or career* or vacanc*) adj5 (retention or retain* or remain* or stay* or leav* or quit* or resign* or attrition or turnover)).tw.	76303
S9	7 or 8	82671

S10	6 and 9	559

**Table 3. MIDRS search terms**		
**Search ID**	**Search terms**	**Results**
S1	Midwi*.tw.	837
S2	(maternity adj3 service*).tw.	19
S3	((work* or profession* or employ* or occupation* or role* or organisation* or position or career* or vacanc*) adj5 (retention or retain* or remain* or stay* or leav* or quit* or resign* or attrition or turnover)).tw.	473
S4	1 or 2	856
S5	3 and 4	39

**Table 4. Scopus search terms**		
**Search ID**	**Search terms**	**Results**
S1	TITLE-ABS-KEY (midwi*) OR TITLE-ABS-KEY (maternity W/5 service*))	57,149
S2	TITLE-ABS-KEY ((work* OR profession* OR employ* OR occupation* OR role* OR organisation* OR position OR career* OR vacanc*) W/5 (retention OR retain* OR remain* OR stay* OR leav* OR quit* OR resign* OR attrition OR turnover))	205,452
S3	((TITLE-ABS-KEY (midwi*) OR TITLE-ABS-KEY (maternity W/5 service*))) AND (TITLE-ABS-KEY ((work* OR profession* OR employ* OR occupation* OR role* OR organisation* OR position OR career* OR vacanc*) W/5 (retention OR retain* OR remain* OR stay* OR leav* OR quit* OR resign* OR attrition OR turnover)))	607
