# Supplementary material for: Mapping factors that may influence attrition and retention of midwives: a scoping review protocol
Source: BMJ Open. 2023 Oct 20;13(10):e076686. doi: 10.1136/bmjopen-2023-076686 (PMC10603492; doi:10.1136/bmjopen-2023-076686)
Supplement: Author's manuscript [file bmjopen-2023-076686.draft_revisions.pdf]

BMJ Open is committed to open peer review. As part of this commitment we make the peer review history of every article we publish publicly available.

When an article is published we post the peer reviewers' comments and the authors' responses online. We also post the versions of the paper that were used during peer review. These are the versions that the peer review comments apply to.

The versions of the paper that follow are the versions that were submitted during the peer review process. They are not the versions of record or the final published versions. They should not be cited or distributed as the published version of this manuscript.

BMJ Open is an open access journal and the full, final, typeset and author-corrected version of record of the manuscript is available on our site with no access controls, subscription charges or pay-per-view fees (http://bmjopen.bmj.com).

If you have any questions on BMJ Open's open peer review process please email info.bmjopen@bmj.com

BMJ Open

# Mapping factors that may influence attrition and retention of midwives: a scoping review protocol

| | |
|---|---|
| Journal: | *BMJ Open* |
| Manuscript ID | bmjopen-2023-076686 |
| Article Type: | Protocol |
| Date Submitted by the Author: | 14-Jun-2023 |
| Complete List of Authors: | Moncrieff, Gill; University of Stirling, Nursing, Midwifery and Allied Health Professions Research Unit<br>Cheyne, Helen; University of Stirling, NMAHP Research Unit; University of Stirling,<br>Downe, Soo; University of Central Lancashire, research in childbirth and health<br>Maxwell, Margaret; University of Stirling, Nursing, Midwifery and Allied Health Professions Research Unit |
| Keywords: | OBSTETRICS, Maternal medicine < OBSTETRICS, Health Services Accessibility |
| | |

SCHOLARONE™
Manuscripts

# Mapping factors that may influence attrition and retention of midwives: a scoping review protocol

**Authors**

Gill Moncrieff[1]

Helen Cheyne[1]

Soo Downe[2]

Margaret Maxwell[1]

* Corresponding author: gill.moncrieff@stir.ac.uk

Affiliations

1. Nursing, Midwifery and Allied Health Professions Research Unit, University of Stirling, Stirling, United Kingdom
2. Research in Childbirth and Health Group, THRIVE Centre, University of Central Lancashire, Preston, United Kingdom

For peer review only

# Mapping factors that may influence attrition and retention of midwives: a scoping review protocol


## Abstract

### Introduction

An appropriately staffed midwifery workforce is essential for the provision of safe and high-quality maternity care. However, there is a global and national shortage of midwives. Understaffed maternity services are frequently identified as contributing to unsafe care provision and adverse outcomes for mothers and babies. While there is a need to recruit midwives through pre-registration midwifery programmes, this is associated with cost and resource implications, and is counteracted to a large extent by the high number of midwives leaving the workforce. It is increasingly recognised that there is a critical need to attend to retention in midwifery in order to develop and maintain safe staffing levels. The objective of this review is to collate and map factors that have been found to influence attrition and retention in midwifery.

### Methods and analysis

Joanna Briggs Institute guidance for scoping reviews and the Preferred Reporting Items for Systematic Reviews and Meta-Analyses extension for Scoping Reviews will be used to guide the review process and reporting of the review. CINAHL, MEDLINE, PsycINFO, and Scopus databases will be used to carry out the search for relevant literature. Results will be screened against inclusion criteria. Data will be extracted using a pre-formed data extraction tool and findings will be presented in narrative, tabular, and graphical formats.

### Ethics and dissemination

The review will collate data from existing research, therefore ethics approval is not required. Findings will be published in journals, presented at conferences, and will be translated into infographics and other formats for online dissemination.


### Strengths and limitations of this study

- This will be the first review to systematically map the factors found to influence midwives' decision to stay in or leave their role as a midwife
- Scoping reviews provide a rigorous and structured method through which to collate and map evidence on a given topic
- This protocol and the full review will follow Joanna Briggs Institute guidance for scoping reviews and will be reported in line with the Preferred Reporting Items for Systematic Reviews and Meta-Analyses extension for Scoping Reviews
- The review will be of relevance to other high income countries but is unlikely be relevant for low and middle income countries

For peer review only

## MAIN SECTION

### Introduction

The provision of safe, effective, and quality maternity services is essential for the health and wellbeing of women and babies[1]. A midwifery workforce that is appropriately and sustainably staffed is integral to this. However, there is a national and global shortage of healthcare professionals, with nurses and midwives at the top of the list among the healthcare professions, representing over 50% of the global shortage[2]. For maternity care, staff shortages appear increasingly to impact on safety.

Several recent maternity investigations and reviews from the United Kingdom (UK) have identified understaffing as a contributary cause in adverse outcomes for mothers and babies[3,4,5,6]. The Care Quality Commission (CQC) frequently find insufficient staffing in maternity units, which they report is putting mothers and babies at risk[7,8,9]. Both the recruitment and retention of staff are contributing to this problem. A primary action outlined in the recent Ockenden review of maternity services at Shrewsbury and Telford Hospital NHS Trust is to ensure sufficient staffing for the provision of safe and sustainable maternity systems[6].

In 2021, the Health and Social Care Committee (HSCC) reported that the NHS in England is short of 1932 midwives, describing staffing shortages in maternity as a persistent problem[10]. While there is a need to recruit new midwives to ameliorate this, training, recruitment, and selection processes come with considerable time and cost implications. Since the HSCC report, the number of midwives in England has fallen by a further 633 full time equivalent posts between April 2021 and April 2022. This is reportedly the largest annual loss of midwifery staff from the NHS since 2009, when records for this measure were first recorded[7].

Historically, the UK has relied on bringing new health professionals into the workforce to deal with staff shortages, whether through educating new health professionals, or looking to international recruitment[11,12]. However, there are ethical issues associated with international recruitment[13], and education and training packages are required due to differing training practices between countries. Moreover, staff from minority ethnic groups have received poor treatment in the past[12,14]. This, along with the UK's exit from the European Union may encourage foreign-trained professionals to choose other countries rather than the UK. Undergraduate training for health professionals in the UK is associated with significant costs[15] and places on courses are finite, particularly given staffing shortages both in practice and educational establishments. Furthermore, applications for places on Nursing and Midwifery course have fallen over recent years and attrition from nursing and midwifery degrees is significant (at 24% and 21% of the student intake respectively)[16].

Equally important, loss of staff from the existing workforce results in the loss of valuable experience. It also has cost and resource implications and reduces productivity and quality of care of care provision[17]. The NHS in general is experiencing ongoing and increasing difficulty, in many areas, with retaining its existing staff, a process that is critical to the effective functioning and sustainability of any organisation[16,18]. Retention also represents a faster and less costly way to maintain the workforce than relying on new recruits. The need for a focus on retention and the development of strategies to increase retention for healthcare workers is increasingly recognised as critical both to attend to the current staffing crises and to facilitate long-term stability and productivity of the healthcare workforce[10,16,18]. This may be increasingly necessary following the experiences of staff over the COVID-19 pandemic, which may have tipped the balance further towards an exodus of staff from the service[18,19].

For peer review only

The objective of the review is to collate and map the factors that have been found to influence attrition and retention in midwifery. It forms part of a larger project funded by the Scottish Government Chief Scientist Office, that is designed to develop a strategy to increase retention within the UK midwifery workforce (the REMAIN study). Following completion of the review, the findings will be used to collaborate with the REMAIN stakeholder groups to identify key questions for subsequent stages of the research and to feed into development of the retention strategy.

The social ecological model developed by McLeroy[20] will be used as a framework for analysis and presentation of findings. An ecological approach to the analysis is systems-oriented, facilitating consideration of the role of the causal processes operating in and across the different system levels, and the relationships within and between these[21]. This moves the focus away from individual causes of problems towards multifactor environmental causes, and thus multilevel systems-focused solutions.

It is recognised that intention to leave and to intention to stay are not mirror constructs, and that influences on intention may differ from factors that influence the act of leaving[18,22]. Furthermore, the decision to stay or leave may include changing role, changing organisation, or leaving the profession altogether[22]. Therefore, analysis and the resulting framework will separate out these constructs where this is possible.

A scoping review was considered appropriate for this review which does not aim to synthesise the findings, rather the objective is to collate and map the factors identified as influencing attrition and retention and present these findings in a clearly illustrated tabular and/or graphical format. Scoping review methodology provides a rigorous and structured approach through which to achieve this [23,24].

**Review questions**

The main research question is:

- What factors influence midwives' intention or decision to stay or leave

Secondary research questions are:

- What associated recommendations have been made to improve retention in midwifery
- What gaps need to be filled to make recommendations for research, policy and practice

**Review registration**

This review protocol has been registered with Open Science Framework.

**Methods and analysis**

The review will be carried out according to the Joanna Briggs Institute (JBI) guidance for scoping reviews[24] and will be structured according to the Preferred Reporting Items for Systematic Reviews and Meta-Analyses (PRISMA) extension for Scoping Reviews[23], both of which have guided the reporting of this protocol.

A preliminary search of PROSPERO and CINAHL search (18/05/2023) confirmed that there are currently no existing or in progress systematic or scoping reviews that collate the factors that influence midwives' motivation to stay in or leave their role.

For peer review only

### Information sources and searches

An initial limited search of MEDLINE was carried out to identify relevant articles to develop the full search strategy. Search terms for the full search strategy were identified based on the titles, abstracts, and index terms used to describe the articles. The search strategy for the initial database was then developed and tested with an information specialist. Table 1 outlines the search strategy developed for CINAHL (via EBSCO). This will be adapted for each of the databases to be used in the full review. A second full search will then be carried out using CINAHL, PsycINFO, and Scopus databases. A third search will be carried out through screening the reference lists of all papers included in the review. Finally, the websites of relevant professional bodies will be searched to identify any relevant grey literature. As stated in JBI guidance, it is possible that additional keywords, search terms or information sources may be identified as the search commences[24]. If this is the case, amendments to the search strategy will be made transparent in the full review.

**Table 1. Search terms**

| Search ID | Search terms | Results |
|---|---|---|
| S1 | (MH "Midwives+") OR (MH "Midwife Attitudes") OR (MH "Midwifery+") OR (MH "Nurse Midwifery") OR (MH "Midwifery Service+") OR (MH "Nurse-Midwifery Service") OR (MH "Maternal Health Services+") | 53,880 |
| S2 | TI ( midwi* or (maternity N3 service*) OR AB ( midwi* or (maternity N3 service*) | 41,052 |
| S3 | (MH "Personnel Retention") OR (MH "Personnel Loyalty") OR (MH "Employment Termination") | 15,649 |
| S4 | TI ( work* or profession* or employ* or occupation* or role* or organisation* or position or career* or vacanc* ) N5 (retention or retain* or remain* or stay* or leav* or quit* or resign* or attrition or turnover ) OR AB ( work* or profession* or employ* or or occupation* or role* or organisation* or position or career* or vacanc*) N5 (retention or retain* or remain* or stay* or leav* or quit* or resign* or attrition or turnover) | 23,722 |
| S5 | S3 OR S4 | 37,028 |
| S6 | S1 OR S2 | 57,065 |
| S7 | S5 AND S6 | 916 |

For peer review only

### Eligibility criteria

JBI guidance defines eligibility according to participants, concept, and context[23].

**Participants**: midwives as defined by the International Confederation of Midwives[25]. This includes midwives that have practiced or practice within a healthcare, education, research, or policy setting, and privately practicing and independent midwives. Where publications include both nurses and midwives, and data for midwives can be disaggregated, these will be included. However, if responses from midwives cannot be separated, these publications will not be included.

**Concept**: factors that influence midwives' intention or decision to stay in or leave their role as a midwife. This will include factors that influence whether midwives move from one role or organisation to another, as well as factors that influence the intention or decision to leave the profession entirely. Only research where the primary focus is decision to stay or leave will be included. Research that has another focus, but that may have decision to stay or leave as an outcome (e.g., research focused on wellbeing), will be excluded.

**Context**: High income counties as classified by the World Bank[26]. Studies from low and middle income countries will be excluded, to identify experiences and perspectives that are similar to the UK maternity context. It is recognised that even with this restriction, contexts that are felt by the review team to be significantly different to the UK may be eligible for inclusion. Where this is the case, any distinctions will be included in the analysis and documented in the findings.

**Types of studies**: all primary (qualitative, quantitative, and mixed methods) research studies will be eligible for inclusion. Reviews will not be eligible for inclusion to avoid duplication of the included studies, but their reference lists will be screened for relevant primary research papers. Relevant grey literature will also be included, for example surveys carried out by professional bodies that may not have been published in journals. Conference abstracts and other non-full text publications will not be eligible for inclusion. There will be no language or date restrictions on the search. Google translate will be used for translation of any non-English language publications.

**Study screening and selection**

Following the database search, the retrieved citations will be uploaded to Rayyan, and duplicates removed. Citations will be screened initially by title and abstract, then by full text using the inclusion/exclusion criteria (table 2). Where articles are excluded at the full text stage, the reason will be recorded on Rayyan and documented in the full review. To ensure consistency within the review team regarding inclusion/exclusion criteria, 20% of the retrieved citations will be screened in duplicate at both the title/abstract and full text stage. Any disagreements will be discussed within the team. The first author will screen the remaining results once consensus has been reached. The screening and selection process will be reported in a Preferred Reporting Items for Systematic Reviews and Meta-Analyses-Scoping Reviews flow diagram.

**Table 2**. Eligibility criteria

| | Inclusion | Exclusion |
|---|---|---|
| Participant | Midwives that practice or have practiced in:<br>- healthcare (including privately practicing/independent midwives)<br>- education<br>- policy<br>- research | Publications that include nurses and midwives, where data for midwives cannot be disaggregated |
| Concept | Decision or intention to:<br>- stay in or change role<br>- stay in or change organisation<br>- stay in or leave the profession | Publications where decision to stay in or leave midwifery is not the primary focus of the research |
| Context | High income countries | Middle income countries<br>Low income countries |

For peer review only

**Data extraction**

Once the articles for inclusion have been selected, data will be extracted onto an Excel document by the first author, using a data extraction tool developed for the purposes of this review (supplementary file). This tool may be modified as necessary as data are extracted. If this does occur, amendments will be detailed in the full review. The following details will be extracted: authors; publication date; title; location of study; aims/objectives; number of participants; study design; factors found to influence the decision to stay or leave; and recommendations for policy, research or practice. The data extraction tool has been developed in collaboration with the review team and the first author will discuss any queries, concerns, or potential modifications during the data extraction process with the rest of team.

Critical appraisal of included studies is not required or usually included as part of the review process for scoping reviews[24]. This is due to the stated purpose of describing and mapping the evidence, rather than making analytical comparisons and/or producing evidence to directly inform practice.

**Data analysis and presentation**

Extracted data will be reviewed and discussed by the review team. Data will be summarised narratively and in a tabular and graphical format. These will focus on the main objective of the review, to summarise and illustrate factors found to influence attrition and retention. Recommendations for research and practice and gaps in the research will also be documented.

**Patient and public involvement**

The REMAIN project includes collaboration with staff, service user, and advisory stakeholder groups. Through stakeholder engagement, the results of the review will be utilised to identify any gaps to be explored as an integral component of the project and will feed into the development of a retention strategy for midwives.

**Ethics and dissemination**

The review will collate data from existing research, therefore ethics approval is not required. Findings will be published in journals, presented at conferences, and will be translated into infographics and other formats for online dissemination.

**Implications**

Sustainable staffing levels are integral to the provision of safe and quality maternity care. This requires appropriate retention of midwives within the workforce. This will be the first review to systematically map the factors that have been found to influence midwives' decision to stay in or leave their role and will inform the development of a strategy to increase retention in midwifery. It is envisaged that findings of this review will be of value to other high income settings, however, a review with different inclusion criteria will be required for low and middle income settings.

For peer review only

## References

1. Renfrew MJ, McFadden A, Bastos MH, Campbell J, Channon AA, Cheung NF, et al. Midwifery and quality care: findings from a new evidence-informed framework for maternal and newborn care. The Lancet. 2014 Sep;384(9948):1129–45
2. WHO. Nursing and midwifery; 2022. Available from: https://www.who.int/news-room/fact-sheets/detail/nursing-and-midwifery
3. Kirkup B. The report of the Morecambe Bay investigation. Department of Health; 2015.
4. Draper E, Kurinczuk J, Kenyon S (Eds) on behalf of MBRRACE-UK. MBRRACE-UK 2017 Perinatal Confidential Enquiry: Term, singleton, intrapartum stillbirth and intrapartum-related neonatal death. University of Leicester; 2017.
5. Knight M, Nair M, Tuffnell D, ShakespeareJ, Kenyon S, Kurinczuk JJ (Eds.) on behalf of MBRRACE-UK. Saving Lives, Improving Mothers' Care: Lessons learned to inform maternity care from the UK and Ireland Confidential Enquiries into Maternal Deaths and Morbidity 2013–15. National Perinatal Epidemiology Unit, Oxford; 2017
6. Ockenden Independent Maternity Review. Final findings, conclusions and essential actions from the Ockenden review of maternity services at Shrewsbury and Telford Hospital NHS Trust; 2022
7. CQC. Areas of specific concern. Care Quality Commission; 2023. Available from: https://www.cqc.org.uk/publication/state-care-202122/concern
8. CQC. CQC rates Surrey maternity service inadequate. Care Quality Commission; 2023. Available from: https://www.cqc.org.uk/press-release/cqc-rates-surrey-maternity-service-inadequate
9. CQC. CQC rates Hertfordshire hospital's maternity service inadequate and serves a warning notice. Care Quality Commission; 2023. Available from: https://www.cqc.org.uk/press-release/cqc-rates-hertfordshire-hospitals-maternity-service-inadequate-and-serves-warning
10. HSCC. The Health and Social Care Committee's Expert Panel: Evaluation of the Government's progress against its policy commitments in the area of maternity services in England. Health and Social Care Committee; 2021. Available from: https://committees.parliament.uk/publications/6560/documents/71747/default/
11. Buchan J. International Recruitment of Nurses: Policy and Practice in the United Kingdom. Health Services Research. 2007 Jun;42(3p2):1321–35.
12. Anderson M, O'Neill C, Macleod Clark J, Street A, Woods M, Johnston-Webber C, et al. Securing a sustainable and fit-for-purpose UK health and care workforce. The Lancet. 2021 May;397(10288):1992–2011.
13. Miller A. NHS global recruitment must not come at the expense of poorer countries, charity warns. BMJ. 2019 Jul 8;l4588.
14. Alexis O. Internationally recruited nurses' experiences in England: A survey approach. Nursing Outlook. 2015 May;63(3):238–44.
15. Jones K, Burns A. Unit Costs of Health and Social Care. University of Kent; 2021. Available from: https://www.pssru.ac.uk/project-pages/unit-costs/unit-costs-of-health-and-social-care-2021/
16. Buchan J, Charlesworth A, Gershlick B, Seccombe I. A critical moment: NHS staffing trends, retention and attrition. The Health foundation; 2019.
17. Buchan J, Shaffer F, Catton H. Policy Brief: Nurse Retention. International Centre on Nurse Migration; 2018.

18. Weyman A, Glendinning R, O'Hara R, Coster J, Roy D, Nolan P. Should I stay or should I go? NHS staff retention in the post COVID-19 world: Challenges and prospects. Institute for Policy Research; 2023.
19. Cordey S, Moncrieff G, Cull J, Sarian A. "There's only so much you can be pushed": magnification of the maternity staffing crisis by the 2020/21 COVID-19 pandemic. BJOG: An International Journal of Obstetrics & Gynaecology. 2022 May 11;129(8).
20. McLeroy KR, Bibeau D, Steckler A, Glanz K. An Ecological Perspective on Health Promotion Programs. Health Education Quarterly [Internet]. 1988 Dec;15(4):351–77. Available from: https://journals.sagepub.com/doi/10.1177/109019818801500401
21. Lounsbury DW, Mitchell SG. Introduction to Special Issue on Social Ecological Approaches to Community Health Research and Action. American Journal of Community Psychology. 2009 Sep 24;44(3-4):213–20.
22. Bolt EET, Winterton J, Cafferkey K. A century of labour turnover research: A systematic literature review. International Journal of Management Reviews. 2022
23. Tricco AC, Lillie E, Zarin W, O'Brien KK, Colquhoun H, Levac D, et al. PRISMA extension for scoping reviews (PRISMA-ScR): Checklist and explanation. Annals of Internal Medicine. 2018 Sep 4;169(7):467–73.
24. Peters MDJ, Godfrey C, McInerney P, Munn Z, Tricco AC, Khalil, H. Chapter 11: Scoping Reviews (2020 version). In: Aromataris E, Munn Z (Editors). JBI Manual for Evidence Synthesis, JBI; 2020. Available from: https://synthesismanual.jbi.global. https://doi.org/10.46658/JBIMES-20-12
25. International Confederation of Midwives. ICM Definitions [Internet]. Internationalmidwives.org. 2019. Available from: https://www.internationalmidwives.org/our-work/policy-and-practice/icm-definitions.html
26. The World Bank. The World by Income and Region. datatopics.worldbank.org. 2021. Available from: https://datatopics.worldbank.org/world-development-indicators/the-world-by-income-and-region.htm

**Supplementary materials**

Data supplement 1

**Author contributions**

GM conceived and designed the study and first draft of this protocol. HC, SD and MM critically reviewed and advised on the first draft. All authors contributed to the final draft of the protocol.

**Funding statement**

This work was supported by the Chief Scientist Office grant number: CAF/23/06

**Acknowledgements**

With thanks to Joshua Cheyne and Catherine Harris for support with formulating the search terms for the review

**Competing interests statement**

The authors have no competing interest to declare

**Word Count**

For peer review only

2073

For peer review only

| Search ID | Search terms | Results |
|---|---|---|
| S1 | (MH "Midwives+") OR (MH "Midwife Attitudes") OR (MH "Midwifery+") OR (MH "Nurse Midwifery") OR (MH "Midwifery Service+") OR (MH "Nurse-Midwifery Service") OR (MH "Maternal Health Services+") | 53,880 |
| S2 | TI ( midwi* or (maternity N3 service*) OR AB ( midwi* or (maternity N3 service*) | 41,052 |
| S3 | (MH "Personnel Retention") OR (MH "Personnel Loyalty") OR (MH "Employment Termination") | 15,649 |
| S4 | TI ( work* or profession* or employ* or occupation* or role* or organisation* or position or career* or vacanc* ) N5 (retention or retain* or remain* or stay* or leav* or quit* or resign* or attrition or turnover ) OR AB ( work* or profession* or employ* or or occupation* or role* or organisation* or position or career* or vacanc*) N5 (retention or retain* or remain* or stay* or leav* or quit* or resign* or attrition or turnover) | 23,722 |
| S5 | S3 OR S4 | 37,028 |
| S6 | S1 OR S2 | 57,065 |
| S7 | S5 AND S6 | 916 |

For peer review only

| **Table 2**. Eligibility criteria | | |
|---|---|---|
| | Inclusion | Exclusion |
| Participant | Midwives that practice or have practiced in:<br>- healthcare (including privately practicing/independent midwives)<br>- education<br>- policy<br>- research | Publications that include nurses and midwives, where data for midwives cannot be disaggregated |
| Concept | Decision or intention to:<br>- stay in or change role<br>- stay in or change organisation<br>- stay in or leave the profession | Publications where decision to stay in or leave midwifery is not the primary focus of the research |
| Context | High income countries | Middle income countries<br>Low income countries |

For peer review only

| Author | Date | Title | Location | Aims/objectives | No. of participants |
|---|---|---|---|---|---|

For peer review only

| Study design | Decision to stay | Decision to leave |
|---|---|---|

For peer review only

| Intention to leave | Recommendations | Other relevant details |
| --- | --- | --- |

For peer review only

BMJ Open

# Mapping factors that may influence attrition and retention of midwives: a scoping review protocol



| | |
|---|---|
| Journal: | *BMJ Open* |
| Manuscript ID | bmjopen-2023-076686.R1 |
| Article Type: | Protocol |
| Date Submitted by the Author: | 29-Aug-2023 |
| Complete List of Authors: | Moncrieff, Gill; University of Stirling, Nursing, Midwifery and Allied Health Professions Research Unit<br>Downe, Soo; University of Central Lancashire, research in childbirth and health<br>Maxwell, Margaret; University of Stirling, Nursing, Midwifery and Allied Health Professions Research Unit<br>Cheyne, Helen; University of Stirling, NMAHP Research Unit; University of Stirling, |
| <b>Primary Subject Heading</b>: | Obstetrics and gynaecology |
| Secondary Subject Heading: | Health policy, Mental health |
| Keywords: | OBSTETRICS, Maternal medicine < OBSTETRICS, MENTAL HEALTH, Burnout, Professional, Health & safety < HEALTH SERVICES ADMINISTRATION & MANAGEMENT, Human resource management < HEALTH SERVICES ADMINISTRATION & MANAGEMENT |
| | |



SCHOLARONE™
Manuscripts

**Mapping factors that may influence attrition and retention of midwives: a scoping review protocol**

**Authors**

Gill Moncrieff[1]

Helen Cheyne[1]

Soo Downe[2]

Margaret Maxwell[1]

* Corresponding author: gill.moncrieff@stir.ac.uk

Affiliations

1. Nursing, Midwifery and Allied Health Professions Research Unit, University of Stirling, Stirling, United Kingdom
2. Research in Childbirth and Health Group, THRIVE Centre, University of Central Lancashire, Preston, United Kingdom

For peer review only

# Mapping factors that may influence attrition and retention of midwives: a scoping review protocol


## Abstract

### Introduction

An appropriately staffed midwifery workforce is essential for the provision of safe and high-quality maternity care. However, there is a global and national shortage of midwives. Understaffed maternity services are frequently identified as contributing to unsafe care provision and adverse outcomes for mothers and babies. While there is a need to recruit midwives through pre-registration midwifery programmes, this has significant resource implications, and is counteracted to a large extent by the high number of midwives leaving the workforce. It is increasingly recognised that there is a critical need to attend to retention in midwifery in order to develop and maintain safe staffing levels. The objective of this review is to collate and map factors that have been found to influence attrition and retention in midwifery.

### Methods and analysis

Joanna Briggs Institute guidance for scoping reviews and the Preferred Reporting Items for Systematic Reviews and Meta-Analyses extension for Scoping Reviews will be used to guide the review process and reporting of the review. CINAHL, MEDLINE, PsycINFO, and Scopus databases will be searched for relevant literature from date of inception to 21 July 2023. Research from high income countries that explores factors that influence leaving intentions for midwives will be included. Literature from low and middle income countries, and studies where nursing and midwifery data cannot be disaggregated will be excluded. Two reviewers will screen 20% of retrieved citations in duplicate, the first author will screen the remaining results. Data will be extracted using a pre-formed data extraction tool by the first author. Findings will be presented in narrative, tabular, and graphical formats.

### Ethics and dissemination

The review will collate data from existing research, therefore ethics approval is not required. Findings will be published in journals, presented at conferences, and will be translated into infographics and other formats for online dissemination.


### Strengths and limitations of this study

- Scoping reviews provide a rigorous and structured method through which to collate and map evidence on a given topic
- This protocol and the full review will follow Joanna Briggs Institute guidance for scoping reviews and will be reported in line with the Preferred Reporting Items for Systematic Reviews and Meta-Analyses extension for Scoping Reviews
- Relevant research that has not been listed in the included databases may not be identified for inclusion in the review
- It may be difficult to establish whether participants intentions to leave or stay relate to changing role, changing organisation, or leaving the profession altogether
- The review will be of relevance to other high income countries but is unlikely be relevant for low and middle income countries

For peer review only

## MAIN SECTION

### Introduction

The provision of safe, effective, and quality maternity services is essential for the health and wellbeing of women and babies (1). Midwives are health professionals that are appropriately educated and registered according to the standards of the International Confederation of Midwives (2), and are integral to sexual, reproductive, maternal, newborn and adolescent health (3). A midwifery workforce that is appropriately and sustainably staffed is integral to this. However, there is a national and global shortage of healthcare professionals, with nurses and midwives at the top of the list among the healthcare professions, representing over 50% of the global shortage (4). For maternity care, staff shortages appear increasingly to impact on safety.

Several recent maternity investigations and reviews from the United Kingdom (UK) have identified understaffing as a contributary cause in adverse outcomes for mothers and babies (5-8). The Care Quality Commission (CQC) frequently find insufficient staffing in maternity units, which they report is putting mothers and babies at risk (9-11). Both the recruitment and retention of staff are contributing to this problem. A primary action outlined in the recent Ockenden review of maternity services at Shrewsbury and Telford Hospital NHS Trust is to ensure sufficient staffing for the provision of safe and sustainable maternity systems (8).

In 2021, the Health and Social Care Committee (HSCC) reported that the NHS in England is short of 1932 midwives, describing staffing shortages in maternity as a persistent problem (12). While there is a need to recruit new midwives to ameliorate this, training, recruitment, and selection processes come with considerable time and cost implications. Since the HSCC report, the number of midwives in England has fallen by a further 633 full time equivalent posts between April 2021 and April 2022. This is reportedly the largest annual loss of midwifery staff from the NHS since 2009, when records for this measure were first recorded (9).

Historically, the UK has relied on bringing new health professionals into the workforce to deal with staff shortages, whether through educating new health professionals, or looking to international recruitment (13,14). However, there are ethical issues associated with international recruitment (15), and education and training packages are required due to differing training practices between countries. Moreover, staff from minority ethnic groups have received poor treatment in the past (14,16). This, along with the UK's exit from the European Union may encourage foreign-trained professionals to choose other countries rather than the UK. Undergraduate training for health professionals in the UK is associated with significant costs and places on courses are finite, particularly given staffing shortages both in practice and educational establishments (17). Furthermore, applications for places on Nursing and Midwifery course have fallen over recent years and attrition from nursing and midwifery degrees is significant (at 24% and 21% of the student intake respectively (18).

Equally important, loss of staff from the existing workforce results in the loss of valuable experience. It also has cost and resource implications and reduces productivity and quality of care of care provision (19). The NHS in general is experiencing ongoing and increasing difficulty, in many areas, with retaining its existing staff, a process that is critical to the effective functioning and sustainability of any organisation (18,20). Retention also represents a faster and less costly way to maintain the workforce than relying on new recruits. The need for a focus on retention and the development of strategies to increase retention for healthcare workers is increasingly recognised as critical both to attend to the current staffing crises and to facilitate long-term stability and productivity of the

For peer review only

healthcare workforce (12,18,20). This may be increasingly necessary following the experiences of staff over the COVID-19 pandemic, which may have tipped the balance further towards an exodus of staff from the service (20,21).

The objective of the review is to collate and map the factors that have been found to influence attrition and retention in midwifery. It forms part of a larger project funded by the Scottish Government Chief Scientist Office, that is designed to develop a strategy to increase retention within the UK midwifery workforce (the REMAIN study). Following completion of the review, the findings will be used to collaborate with the REMAIN stakeholder groups to identify key questions for subsequent stages of the research and to feed into development of the retention strategy.

The social ecological model developed by McLeroy (22) will be used as a framework for analysis and presentation of findings. An ecological approach to the analysis is systems-oriented, facilitating consideration of the role of the causal processes operating in and across the different system levels, and the relationships within and between these (23). This moves the focus away from individual causes of problems towards multifactor environmental causes, and thus multilevel systems-focused solutions.

It is recognised that intention to leave and to intention to stay are not mirror constructs, and that influences on intention may differ from factors that influence the act of leaving (19,24). Furthermore, the decision to stay or leave may include changing role, changing organisation, or leaving the profession altogether (24). Therefore, analysis and the resulting framework will separate out these constructs where this is possible.

A scoping review was considered an appropriate method for this review which does not aim to synthesise the findings, rather the objective is to collate and map the factors identified as influencing attrition and retention and present these findings in a clearly illustrated tabular and/or graphical format. Scoping review methodology provides a rigorous and structured approach through which to achieve this (25,26).

**Review questions**

The main research question is:

- What factors influence midwives' intention or decision to stay or leave?

Secondary research questions are:

- What associated recommendations have been made to improve retention in midwifery?
- What gaps need to be filled to make recommendations for research, policy and practice?

**Review registration**

This review protocol has been registered with Open Science Framework.

**Methods and analysis**

The review will be carried out according to the Joanna Briggs Institute (JBI) guidance for scoping reviews (26) and will be structured according to the Preferred Reporting Items for Systematic Reviews and Meta-Analyses (PRISMA) extension for Scoping Reviews (25), both of which have guided the reporting of this protocol.

For peer review only

A preliminary search of PROSPERO and CINAHL search (18/05/2023) confirmed that there are currently no existing or in progress systematic or scoping reviews that collate the factors that influence midwives' motivation to stay in or leave their role.

**Information sources and searches**

Databases will be searched from date of inception to July 21 2023. An initial limited search of MEDLINE was carried out to identify relevant articles to develop the full search strategy. Search terms for the full search strategy were identified based on the titles, abstracts, and index terms used to describe the articles. The search strategy for the initial database was then developed and tested with an information specialist. Table 1 outlines the search strategy developed for CINAHL (via EBSCO). This will be adapted for each of the databases to be used in the full review. A full search will then be carried out using MEDLINE, MIDRS, and Scopus databases (supplementary file 1). A second search will be carried out through screening the reference lists of all papers included in the review. Finally, the websites of professional bodies will be searched, and relevant professionals will be contacted to identify grey literature for inclusion. As stated in JBI guidance, it is possible that additional keywords, search terms or information sources may be identified as the search commences (26). If this is the case, amendments to the search strategy will be made transparent in the full review.

**Table 1. Search terms**

| Search ID | Search terms | Results |
|---|---|---|
| S1 | (MH "Midwives+") OR (MH "Midwife Attitudes") OR (MH "Midwifery+") OR (MH "Nurse Midwifery") OR (MH "Midwifery Service+") OR (MH "Nurse-Midwifery Service") OR (MH "Maternal Health Services+") | 53,880 |
| S2 | TI ( midwi* or (maternity N3 service*) OR AB ( midwi* or (maternity N3 service*) | 41,052 |
| S3 | (MH "Personnel Retention") OR (MH "Personnel Loyalty") OR (MH "Employment Termination") | 15,649 |
| S4 | TI ( work* or profession* or employ* or occupation* or role* or organisation* or position or career* or vacanc* ) N5 (retention or retain* or remain* or stay* or leav* or quit* or resign* or attrition or turnover ) OR AB ( work* or profession* or employ* or or occupation* or role* or organisation* or position or career* or vacanc*) N5 (retention or retain* or remain* or stay* or leav* or quit* or resign* or attrition or turnover) | 23,722 |
| S5 | S3 OR S4 | 37,028 |
| S6 | S1 OR S2 | 57,065 |
| S7 | S5 AND S6 | 429 |

**Eligibility criteria**

JBI guidance defines eligibility according to participants, concept, and context (26).

**Participants**: midwives as defined by the International Confederation of Midwives (2). This includes midwives that have practiced or practice within a healthcare, education, research, or policy setting, and privately practicing and independent midwives. Where publications include both nurses and

For peer review only - http://bmjopen.bmj.com/site/about/guidelines.xhtml

midwives, and data for midwives can be disaggregated, these will be included. However, if responses from midwives cannot be separated, these publications will not be included.

**Concept**: factors that influence midwives' intention or decision to stay in or leave their role as a midwife. This will include factors that influence whether midwives move from one role or organisation to another, as well as factors that influence the intention or decision to stay in or leave the profession entirely. Only research where the primary focus is decision to stay or leave will be included. Research that has another focus, but that may have decision to stay or leave as an outcome (e.g., research focused on wellbeing), will be excluded. Where studies measure leaving intention quantitatively, but do not explore the associated reasons, these will also be excluded from the analysis.

**Context**: High income countries as classified by the World Bank (27). Studies from low and middle income countries will be excluded, in order to focus on perspectives and experiences that derive from a similar context to that of the UK maternity system. It is recognised that even with this restriction, contexts that are considered to be disparate to the UK may be eligible for inclusion. Where this is the case, any distinctions will be included in the analysis and documented in the findings.

**Types of studies**: all primary (qualitative, quantitative, and mixed methods) research studies will be eligible for inclusion. Reviews will not be eligible for inclusion to avoid duplication of the included studies, but their reference lists will be screened for relevant primary research papers. Relevant grey literature will also be included, for example surveys carried out by professional bodies that may not have been published in journals. Conference abstracts and other non-full text publications will not be eligible for inclusion. There will be no language or date restrictions on the search. Google translate will be used for translation of any non-English language publications.

**Study screening and selection**

Following the database search, the retrieved citations will be uploaded to Rayyan, and duplicates removed. Citations will be screened initially by title and abstract, then by full text using the inclusion/exclusion criteria (table 2). Where articles are excluded at the full text stage, the reason will be recorded on Rayyan and documented in the full review. To ensure consistency within the review team regarding inclusion/exclusion criteria, 20% of the retrieved citations will be screened by two reviewers at both the title/abstract and full text stage. Any disagreements will be discussed within the team. The first author will screen the remaining results once consensus has been reached. The screening and selection process will be reported in a Preferred Reporting Items for Systematic Reviews and Meta-Analyses-Scoping Reviews flow diagram.

**Table 2**. Eligibility criteria

| | Inclusion | Exclusion |
|---|---|---|
| Participant | Midwives that practice or have practiced in:<br>- healthcare (including privately practicing/independent midwives)<br>- education<br>- policy<br>- research | Publications that include nurses and midwives, where data for midwives cannot be disaggregated |

For peer review only

| | | |
|---|---|---|
| Concept | Decision or intention to:<br>- stay in or change role<br>- stay in or change organisation<br>- stay in or leave the profession | Publications where decision to stay in or leave midwifery is not the primary focus of the research |
| Context | High income countries | Middle income countries<br>Low income countries |

**Data extraction**

Once the articles for inclusion have been selected, data will be extracted onto an Excel document by the first author, using a data extraction tool developed for the purposes of this review (supplementary file 2). This tool may be modified as necessary as data are extracted. If this does occur, amendments will be detailed in the full review. The following details will be extracted: authors; publication date; title; location of study; aims/objectives; number of participants; study design; area of midwifery practice; factors found to influence the decision to stay or leave; and recommendations for policy, research or practice. The data extraction tool has been developed in collaboration with the review team and the first author will discuss any queries, concerns, or potential modifications during the data extraction process with the rest of team.

Critical appraisal of included studies is not required or usually included as part of the review process for scoping reviews (26). This is due to the stated purpose of describing and mapping the evidence, rather than making analytical comparisons and/or producing evidence to directly inform practice.

**Data analysis and presentation**

Extracted data will be reviewed and discussed by the review team. Data will be summarised narratively and in a tabular and graphical format. These will focus on the main objective of the review, to summarise and illustrate factors found to influence attrition and retention. Recommendations for research and practice and gaps in the research will also be documented.

**Patient and public involvement**

The REMAIN project includes engagement with staff, service user, and expert members of its Intervention Development Group to inform research processes and refine findings. Through such stakeholder engagement, the findings of the review will be prioritised to feed into the development of a retention strategy for midwives.

**Ethics and dissemination**

The review will collate data from existing research, therefore ethics approval is not required. Findings will be published in journals, presented at conferences, and will be translated into infographics and other formats for online dissemination.

**Discussion**

Sustainable staffing levels are integral to the provision of safe and quality maternity care. This requires appropriate retention of midwives within the workforce. This will be the first review to systematically map the factors that have been found to influence midwives' leaving intentions, providing valuable information to feed into retention-related activities and guidelines for policy and practice. Ultimately it will inform the development of an evidence-based retention programme that aims to reduce attrition within the midwifery workforce.

For peer review only

A scoping review is the appropriate method for this review where the objective is to is to collate and map the factors that have been found to influence attrition and retention in midwifery, rather than synthesising findings or making analytical comparisons (26). Stakeholder engagement with the REMAIN Intervention Development Group will prioritise findings to identify recommendations for practice and to feed into the development of a retention strategy for midwives. This engagement exercise will also identify gaps to be explored as part of the research.

JBI guidance for scoping reviews has been followed to develop this protocol and will be used for the full review, providing a rigorous and structured method to collate and map the evidence relating to midwives leaving intentions (26). It is however recognised that there may be some research, such as workforce surveys carried out by relevant professional bodies, that is not listed in bibliographic databases. To counter this, we will carry out additional searches, including screening reference lists of included publications, searching the websites of professional bodies, and contacting relevant professionals. However, it is possible that relevant research may not be picked up through these additional searches.

In addition, retention is a concept that has yet to be consistently defined and understood, both within the relevant literature, and in research, policy and practice (19,24). As a result, it is likely that it will be challenging to determine whether the included data refers to midwives' intentions to move to another role in the same organisation, or to move to a different organisation, or whether they intend to leave the profession altogether. Where possible however, data for these particular constructs will be separated and clarified in the analysis and findings of the review.

Only data from high income countries will be eligible for inclusion, with the intention of producing findings that are relevant to the UK and other similar maternity systems. However, it is recognised that this distinction is not necessarily clear and that we may as a result include data that relate to divergent maternity systems. The context for each included study will however be recorded and if this issue arises, it will be discussed within the review. A separate review will be required for low and middle income settings.

This review is timely and highly necessary to inform retention-related activities. Working conditions in maternity have been found to have a profound and detrimental impact on the mental health and leaving intentions of midwives, and on the quality and safety of care provision (28,29). As midwives leave, this exacerbates these issues, resulting in a vicious cycle of staff attrition. As a result, there has been considerable loss of valuable experience from the workforce, compromising the safety of care provision, and the clinical education our future midwives (12,30). This situation cannot be resolved simply by adding more midwives. There is a much-needed commitment to attend to the underlying factors that motivate leaving intentions, many of which are modifiable with resolve (29). This review will identify and collate these factors, providing valuable evidence with which to begin this endeavour.

For peer review only

## References


1. Renfrew MJ, McFadden A, Bastos MH, Campbell J, Channon AA, Cheung NF, et al. Midwifery and quality care: findings from a new evidence-informed framework for maternal and newborn care. The Lancet. 2014 Sep;384(9948):1129–45
2. ICM. ICM Definitions. https://www.internationalmidwives.org/assets/files/definitions-files/2023/07/08l_en_international-definition-of-the-midwife.pdf. International Confederation of Midwives; 2019. Available from: https://www.internationalmidwives.org/our-work/policy-and-practice/icm-definitions.html
3. WHO. The state of the world's midwifery 2021; 2021. World Health Organisation. Available from: https://www.unfpa.org/publications/sowmy-2021
4. WHO. Nursing and midwifery; 2022. Available from: https://www.who.int/news-room/fact-sheets/detail/nursing-and-midwifery
5. Kirkup B. The report of the Morecambe Bay investigation. Department of Health; 2015.
6. Draper E, Kurinczuk J, Kenyon S (Eds) on behalf of MBRRACE-UK. MBRRACE-UK 2017 Perinatal Confidential Enquiry: Term, singleton, intrapartum stillbirth and intrapartum-related neonatal death. University of Leicester; 2017.
7. Knight M, Nair M, Tuffnell D, ShakespeareJ, Kenyon S, Kurinczuk JJ (Eds.) on behalf of MBRRACE-UK. Saving Lives, Improving Mothers' Care: Lessons learned to inform maternity care from the UK and Ireland Confidential Enquiries into Maternal Deaths and Morbidity 2013–15. National Perinatal Epidemiology Unit, Oxford; 2017
8. Ockenden Independent Maternity Review. Final findings, conclusions and essential actions from the Ockenden review of maternity services at Shrewsbury and Telford Hospital NHS Trust; 2022
9. CQC. Areas of specific concern. Care Quality Commission; 2023. Available from: https://www.cqc.org.uk/publication/state-care-202122/concern
10. CQC. CQC rates Surrey maternity service inadequate. Care Quality Commission; 2023. Available from: https://www.cqc.org.uk/press-release/cqc-rates-surrey-maternity-service-inadequate
11. CQC. CQC rates Hertfordshire hospital's maternity service inadequate and serves a warning notice. Care Quality Commission; 2023. Available from: https://www.cqc.org.uk/press-release/cqc-rates-hertfordshire-hospitals-maternity-service-inadequate-and-serves-warning
12. HSCC. The Health and Social Care Committee's Expert Panel: Evaluation of the Government's progress against its policy commitments in the area of maternity services in England. Health and Social Care Committee; 2021. Available from: https://committees.parliament.uk/publications/6560/documents/71747/default/
13. Buchan J. International Recruitment of Nurses: Policy and Practice in the United Kingdom. Health Services Research. 2007 Jun;42(3p2):1321–35.


For peer review only

14. Anderson M, O'Neill C, Macleod Clark J, Street A, Woods M, Johnston-Webber C, et al. Securing a sustainable and fit-for-purpose UK health and care workforce. The Lancet. 2021 May;397(10288):1992–2011.

15. Miller A. NHS global recruitment must not come at the expense of poorer countries, charity warns. BMJ. 2019 Jul 8;l4588.

16. Alexis O. Internationally recruited nurses' experiences in England: A survey approach. Nursing Outlook. 2015 May;63(3):238–44.

17. Jones K, Burns A. Unit Costs of Health and Social Care. University of Kent; 2021. Available from: https://www.pssru.ac.uk/project-pages/unit-costs/unit-costs-of-health-and-social-care-2021/

18. Buchan J, Charlesworth A, Gershlick B, Seccombe I. A critical moment: NHS staffing trends, retention and attrition. The Health foundation; 2019.

19. Buchan J, Shaffer F, Catton H. Policy Brief: Nurse Retention. International Centre on Nurse Migration; 2018.

20. Weyman A, Glendinning R, O'Hara R, Coster J, Roy D, Nolan P. Should I stay or should I go? NHS staff retention in the post COVID-19 world: Challenges and prospects. Institute for Policy Research; 2023.

21. Cordey S, Moncrieff G, Cull J, Sarian A. "There's only so much you can be pushed": magnification of the maternity staffing crisis by the 2020/21 COVID-19 pandemic. BJOG: An International Journal of Obstetrics & Gynaecology. 2022 May 11;129(8).

22. McLeroy KR, Bibeau D, Steckler A, Glanz K. An Ecological Perspective on Health Promotion Programs. Health Education Quarterly [Internet]. 1988 Dec;15(4):351–77. Available from: https://journals.sagepub.com/doi/10.1177/109019818801500401

23. Lounsbury DW, Mitchell SG. Introduction to Special Issue on Social Ecological Approaches to Community Health Research and Action. American Journal of Community Psychology. 2009 Sep 24;44(3-4):213–20.

24. Bolt EET, Winterton J, Cafferkey K. A century of labour turnover research: A systematic literature review. International Journal of Management Reviews. 2022

25. Tricco AC, Lillie E, Zarin W, O'Brien KK, Colquhoun H, Levac D, et al. PRISMA extension for scoping reviews (PRISMA-ScR): Checklist and explanation. Annals of Internal Medicine. 2018 Sep 4;169(7):467–73.

26. Peters MDJ, Godfrey C, McInerney P, Munn Z, Tricco AC, Khalil, H. Chapter 11: Scoping Reviews (2020 version). In: Aromataris E, Munn Z (Editors). JBI Manual for Evidence Synthesis, JBI; 2020. Available from: https://synthesismanual.jbi.global. https://doi.org/10.46658/JBIMES-20-12

27. The World Bank. The World by Income and Region. datatopics.worldbank.org. 2021. Available from: https://datatopics.worldbank.org/world-development-indicators/the-world-by-income-and-region.htm

For peer review only

28. Hunter B, Fenwick J, Sidebotham DM, Henley DJ. Midwives in the United Kingdom: Levels of burnout, depression, anxiety and stress and associated predictors. Midwifery [Internet]. 2019 Aug;79(102526). Available from: https://pubmed.ncbi.nlm.nih.gov/31473405/

29. Moncrieff G, Cheyne H, Downe S, Hunter B. Factors that influence midwives' leaving intentions: A moral imperative to intervene. Midwifery. 2023. Available from: https://www.sciencedirect.com/science/article/abs/pii/S0266613823001961

30. APPG on Maternity and Baby Loss Joint Report. 2022. Report of the Baby Loss and Maternity All Party Parliamentary Groups. Safe Staffing: The impact of staffing shortages in maternity and neonatal care. House of Commons. https://commonslibrary.parliament.uk/research-briefings/cdp-2022-0174/

For peer review only

**Supplementary materials**

Data supplement 1

Data supplement 2

**Author contributions**

GM conceived and designed the study and first draft of this protocol. HC, SD and MM critically reviewed and advised on the first draft. All authors contributed to the final draft of the protocol.

**Funding statement**

This work was supported by the Chief Scientist Office, which is part of the Scottish Government Health Directorates. Grant number: CAF/23/06

**Acknowledgements**

With thanks to Joshua Cheyne and Catherine Harris for support with formulating the search terms for the review

**Competing interests statement**

The authors have no competing interest to declare

**Word Count**

2656

**Database searches**

| Table 1. CINAHL search terms | | |
|---|---|---|
| **Search ID** | **Search terms** | **Results** |
| S1 | (MH "Midwives+") OR (MH "Midwife Attitudes") OR (MH "Midwifery+") OR (MH "Nurse Midwifery") OR (MH "Midwifery Service+") OR (MH "Nurse-Midwifery Service") OR (MH "Maternal Health Services+") | 53,880 |
| S2 | TI ( midwi* or (maternity N3 service*) OR AB ( midwi* or (maternity N3 service*) | 41,052 |
| S3 | (MH "Personnel Retention") OR (MH "Personnel Loyalty") OR (MH "Employment Termination") | 15,649 |
| S4 | TI ( work* or profession* or employ* or occupation* or role* or organisation* or position or career* or vacanc* ) N5 (retention or retain* or remain* or stay* or leav* or quit* or resign* or attrition or turnover ) OR AB ( work* or profession* or employ* or or occupation* or role* or organisation* or position or career* or vacanc*) N5 (retention or retain* or remain* or stay* or leav* or quit* or resign* or attrition or turnover) | 23,722 |
| S5 | S3 OR S4 | 37,028 |
| S6 | S1 OR S2 | 57,065 |
| S7 | S5 AND S6 | 429 |

| Table 2. MEDLINE search terms | | |
|---|---|---|
| **Search ID** | **Search terms** | **Results** |
| S1 | Midwifery/ | 21300 |
| S2 | Nurse Midwives/ | 7545 |
| S3 | (exp Maternal Health Services/ | 57972 |
| S4 | Midwi*.tw. | 29057 |
| S5 | (maternity adj3 service*).tw. | 2735 |
| S6 | 1 or 2 or 3 or 4 or 5 | 91332 |
| S7 | personnel loyalty/ or personnel turnover/ or work engagement/ | 8644 |
| S8 | ((work* or profession* or employ* or occupation* or role* or organisation* or position or career* or vacanc*) adj5 (retention or retain* or remain* or stay* or leav* or quit* or resign* or attrition or turnover)).tw. | 76303 |
| S9 | 7 or 8 | 82671 |

For peer review only

| | | |
|---|---|---|
| S10 | 6 and 9 | 559 |

**Table 3. MIDRS search terms**

| Search ID | Search terms | Results |
|---|---|---|
| S1 | Midwi*.tw. | 837 |
| S2 | (maternity adj3 service*).tw. | 19 |
| S3 | ((work* or profession* or employ* or occupation* or role* or organisation* or position or career* or vacanc*) adj5 (retention or retain* or remain* or stay* or leav* or quit* or resign* or attrition or turnover)).tw. | 473 |
| S4 | 1 or 2 | 856 |
| S5 | 3 and 4 | 39 |

**Table 4. Scopus search terms**

| Search ID | Search terms | Results |
|---|---|---|
| S1 | TITLE-ABS-KEY ( midwi* ) OR TITLE-ABS-KEY ( maternity W/5 service* ) ) | 57,149 |
| S2 | TITLE-ABS-KEY ( ( work* OR profession* OR employ* OR occupation* OR role* OR organisation* OR position OR career* OR vacanc* ) W/5 ( retention OR retain* OR remain* OR stay* OR leav* OR quit* OR resign* OR attrition OR turnover ) ) | 205,452 |
| S3 | ( ( TITLE-ABS-KEY ( midwi* ) OR TITLE-ABS-KEY ( maternity W/5 service* ) ) ) AND ( TITLE-ABS-KEY ( ( work* OR profession* OR employ* OR occupation* OR role* OR organisation* OR position OR career* OR vacanc* ) W/5 ( retention OR retain* OR remain* OR stay* OR leav* OR quit* OR resign* OR attrition OR turnover ) ) ) | 607 |

For peer review only

**Data Extraction: Mapping factors that influence midwives leaving intetntions**

| Author | Date | Title | Location | Aims/objectives | No. of participants | Study design | Area of midwifery practice | Decision to stay | Decision to leave | Intention to leave | Recommendations | Other relevant details |
|---|---|---|---|---|---|---|---|---|---|---|---|---|

For peer review only

## Preferred Reporting Items for Systematic reviews and Meta-Analyses extension for Scoping Reviews (PRISMA-ScR) Checklist

| SECTION | ITEM | PRISMA-ScR CHECKLIST ITEM | REPORTED ON PAGE # |
|---|---|---|---|
| **TITLE** | | | |
| Title | 1 | Identify the report as a scoping review. | Page 2, line 1 |
| **ABSTRACT** | | | |
| Structured summary | 2 | Provide a structured summary that includes (as applicable): background, objectives, eligibility criteria, sources of evidence, charting methods, results, and conclusions that relate to the review questions and objectives. | Page 2, lines 5 - 31 |
| **INTRODUCTION** | | | |
| Rationale | 3 | Describe the rationale for the review in the context of what is already known. Explain why the review questions/objectives lend themselves to a scoping review approach. | Context: pages 3 – 4<br>Scoping review rationale: Page 4, lines 29 - 33 |
| Objectives | 4 | Provide an explicit statement of the questions and objectives being addressed with reference to their key elements (e.g., population or participants, concepts, and context) or other relevant key elements used to conceptualize the review questions and/or objectives. | Objective: page 4, line 21<br>Questions: page 4 lines 34 - 39 |
| **METHODS** | | | |
| Protocol and registration | 5 | Indicate whether a review protocol exists; state if and where it can be accessed (e.g., a Web address); and if available, provide registration information, including the registration number. | N/A |
| Eligibility criteria | 6 | Specify characteristics of the sources of evidence used as eligibility criteria (e.g., years considered, language, and publication status), and provide a rationale. | Page 6, lines 1 - 38 |
| Information sources* | 7 | Describe all information sources in the search (e.g., databases with dates of coverage and contact with authors to identify additional sources), as well as the date the most recent search was executed. | Page 45, lines 11 -23 |
| Search | 8 | Present the full electronic search strategy for at least 1 database, including any limits used, such that it could be repeated. | Page 5, line 24 |
| Selection of sources of evidence† | 9 | State the process for selecting sources of evidence (i.e., screening and eligibility) included in the scoping review. | Page 6, line 29 - 38 |
| Data charting process‡ | 10 | Describe the methods of charting data from the included sources of evidence (e.g., calibrated forms or forms that have been tested by the team before their use, and whether data charting was done independently or in duplicate) and any processes for obtaining and confirming data from investigators. | Page 7, lines 2 - 11 |
| Data items | 11 | List and define all variables for which data were sought and any assumptions and simplifications made. | Page 7, lines 6 - 9 |
| Critical appraisal of individual sources of evidence§ | 12 | If done, provide a rationale for conducting a critical appraisal of included sources of evidence; describe the methods used and how this information was used | N/A |

For peer review only

| SECTION | ITEM | PRISMA-ScR CHECKLIST ITEM | REPORTED ON PAGE # |
|---|---|---|---|
| | | in any data synthesis (if appropriate). | |
| Synthesis of results | 13 | Describe the methods of handling and summarizing the data that were charted. | Page 7, lines 15 - 19 |
| **RESULTS** | | | |
| Selection of sources of evidence | 14 | Give numbers of sources of evidence screened, assessed for eligibility, and included in the review, with reasons for exclusions at each stage, ideally using a flow diagram. | N/A |
| Characteristics of sources of evidence | 15 | For each source of evidence, present characteristics for which data were charted and provide the citations. | N/A |
| Critical appraisal within sources of evidence | 16 | If done, present data on critical appraisal of included sources of evidence (see item 12). | N/A |
| Results of individual sources of evidence | 17 | For each included source of evidence, present the relevant data that were charted that relate to the review questions and objectives. | N/A |
| Synthesis of results | 18 | Summarize and/or present the charting results as they relate to the review questions and objectives. | N/A |
| **DISCUSSION** | | | |
| Summary of evidence | 19 | Summarize the main results (including an overview of concepts, themes, and types of evidence available), link to the review questions and objectives, and consider the relevance to key groups. | N/A |
| Limitations | 20 | Discuss the limitations of the scoping review process. | N/A |
| Conclusions | 21 | Provide a general interpretation of the results with respect to the review questions and objectives, as well as potential implications and/or next steps. | N/A |
| **FUNDING** | | | |
| Funding | 22 | Describe sources of funding for the included sources of evidence, as well as sources of funding for the scoping review. Describe the role of the funders of the scoping review. | Included sources: N/A Funder: page 11, lines 8 and 9 |

JBI = Joanna Briggs Institute; PRISMA-ScR = Preferred Reporting Items for Systematic reviews and Meta-Analyses extension for Scoping Reviews.
* Where *sources of evidence* (see second footnote) are compiled from, such as bibliographic databases, social media platforms, and Web sites.
† A more inclusive/heterogeneous term used to account for the different types of evidence or data sources (e.g., quantitative and/or qualitative research, expert opinion, and policy documents) that may be eligible in a scoping review as opposed to only studies. This is not to be confused with *information sources* (see first footnote).
‡ The frameworks by Arksey and O'Malley (6) and Levac and colleagues (7) and the JBI guidance (4, 5) refer to the process of data extraction in a scoping review as data charting.
§ The process of systematically examining research evidence to assess its validity, results, and relevance before using it to inform a decision. This term is used for items 12 and 19 instead of "risk of bias" (which is more applicable to systematic reviews of interventions) to include and acknowledge the various sources of evidence that may be used in a scoping review (e.g., quantitative and/or qualitative research, expert opinion, and policy document).

*From:* Tricco AC, Lillie E, Zarin W, O'Brien KK, Colquhoun H, Levac D, et al. PRISMA Extension for Scoping Reviews (PRISMAScR): Checklist and Explanation. Ann Intern Med. 2018;169:467–473. doi: 10.7326/M18-0850.


For peer review only - http://bmjopen.bmj.com/site/about/guidelines.xhtml
